# The Health Initiative Program for Kids (HIP Kids): effects of a 1-year multidisciplinary lifestyle intervention on adiposity and quality of life in obese children and adolescents - a longitudinal pilot intervention study

**DOI:** 10.1186/s12887-014-0296-1

**Published:** 2014-12-05

**Authors:** Dirk E Bock, Tracy Robinson, Jamie A Seabrook, Meghan Rombeek, Kambiz Norozi, Guido Filler, Ralf Rauch, Cheril L Clarson

**Affiliations:** Department of Pediatrics, Children’s Hospital, London Health Sciences Centre, Western University, 800 Commissioners Road East, London, ON N6A 5W9 Canada; Division of Pediatric Cardiology and Intensive Care Medicine, Hannover Medical School, Carl-Neuberg Str. 1, 30625 Hannover, Germany; Lawson Health Research Institute, 750 Base Line Road, Suite 300, London, ON N6C 2R5 Canada; Children’s Health Research Institute, 800 Commissioners Road East, London, ON N6C 2V5 Canada; Rems Murr Kliniken, Department of Pediatrics, Winnender Str. 45, 71334 Waiblingen, Germany; Division of Food & Nutritional Sciences, Brescia University College, Western University, 1285 Western Rd, London, ON N6G 1H2 Canada

**Keywords:** Obesity, Overweight, Behavior modification, Family-centered, BMI z-score, Nutrition, Pediatric, Outpatient, Childhood, Youth

## Abstract

**Background:**

Though recent data suggest that multidisciplinary outpatient interventions can have a positive effect on childhood obesity, it is still unclear which program components are most beneficial and how they affect quality of life (QoL). The aim of this study was to determine if a 1-year multidisciplinary, family-centered outpatient intervention based on social cognitive theory would be effective in (i) preventing further increases in BMI and BMI z-score, and (ii) improving QoL in obese children and adolescents.

**Methods:**

Obese children and adolescents 8–17 years of age and their families participated in this 1-year longitudinal pilot intervention study. The intervention consisted of fifteen 90-minute educational sessions led by a dietitian, exercise specialist, and social worker. Anthropometric measures, body composition, and QoL (Pediatric Quality of Life Inventory 4.0), were assessed at baseline, 3 months, and 12 months. Laboratory values were measured at baseline and 12 months. The primary outcome measures were change in BMI and BMI z-score, secondary outcome measures included change in QoL and body composition. A paired sample *t*-test was used to assess within-group differences and 95% confidence intervals were reported for the mean differences.

**Results:**

42 obese children and adolescents (21 girls) completed the 1-year intervention (mean age 12.8 ± 3.14 years). Mean baseline BMI was 31.96 ± 5.94 kg/m^2^ and BMI z-score was +2.19 ± 0.34. Baseline QoL (self-assessments and parental assessments) was impaired: mean baseline scores were 74.5 ± 16.5 and 63.7 ± 19.4 for physical functioning and 69.0 ± 14.9 and 64.0 ± 18.3 for emotional functioning, respectively. At 12 months, BMI z-score had decreased (−0.07 ± 0.11, 95% CI: −0.11 to −0.04). BMI (0.80 ± 1.57 kg/m^2^, 95% CI 0.31 to 1.29) and fat-free mass (4.02 ± 6.27 kg, 95% CI 1.90 to 6.14) increased, but % body fat and waist circumference did not. Both the parent-reported physical (11.3 ± 19.2, 95% CI 4.7 to 17.9) and emotional (7.7 ± 15.7, 95% CI 2.3 to 13.0) functioning QoL scores and the children's self-reported physical (5.3 ± 17.1, 95% CI 0.5 to 11.1) and emotional (7.9 ± 14.3, 95% CI 3.2 to 12.7) functioning scores significantly improved.

**Conclusions:**

Following a 1-year intervention, the participants’ BMI z-scores and QoL improved, while other adiposity-related measures of body composition remained unchanged.

**Trial registration:**

UMIN Clinical Trials Registry UMIN000015622.

**Electronic supplementary material:**

The online version of this article (doi:10.1186/s12887-014-0296-1) contains supplementary material, which is available to authorized users.

## Background

The rate of childhood obesity in North America has more than tripled over the past 30 years: approximately 32% of North American children are now overweight and between 11.7% and 16.9% are obese [1,2]. Many major physical and psychological comorbidities are associated with childhood obesity, including hypertension, dyslipidemia, impaired glucose tolerance, obstructive sleep apnea, and depression [3-5]. Studies have also shown that quality of life (QoL) in this population is impaired [6,7]. Moreover, the majority of those obese during adolescence remain so through adulthood, and this adverse metabolic profile results in sequelae that impose major health and economic challenges on both the individual and society [8,9].

Studies implementing a comprehensive set of multidisciplinary lifestyle interventions that focus on the whole family and incorporate changes in diet, physical activity, and behavior have been found to reduce the degree of obesity in affected children [10-12]. Nevertheless, the availability and accessibility of such interventions is limited, particularly at a community level, and their effects vary substantially, depending on the design of the intervention, the participants’ age, the level of parental involvement, and other factors [10].

In 2008, the Provincial Council for Children’s Health (PCCH) in Ontario, Canada, identified a severe lack of comprehensive secondary and tertiary care treatment programs for children and adolescents with moderate or severe obesity-related morbidities and for those who had been unsuccessful with primary care management. In their proposed framework, the PCCH suggested comparing an interventional model incorporating structure and resources for effective secondary and tertiary care interventions with the anticipated long-term health consequences and related health care costs associated with not providing such an intervention [PCCH Expert Panel for Child and Youth Overweight and Obesity, Provincial Council for Maternal and Child Health. A Proposed Service Delivery Framework for Healthy Weights for Children and Youth across the Continuum of Care: Toronto, 2008 - unpublished data].

The Health Initiative Program for Kids (HIP Kids) was developed both to address this report and to create such an intervention in southwestern Ontario where, despite being in high demand, no such interventions existed. The program follows a pilot intervention model and merges the gap between the needs of obese children and adolescents and their families and the medical and research community. The aim of this research was to prospectively evaluate whether HIP Kids, a 1-year multidisciplinary, family-centered hospital outpatient clinic-based intervention for obese children and adolescents and their families, would be effective in preventing further increases in body mass index (BMI) and BMI standard deviation score (BMI z-score, BMI-Z) and in improving QoL. We hypothesized that the HIP Kids program will i) prevent further increases in BMI and BMI-Z, and ii) increase standardized QoL scores in intervention subjects over the study period.

## Methods

### Design and study population

Between March 2009 and January 2011, family physicians and pediatricians in London, Ontario and surrounding communities were invited to refer obese children and adolescents to the HIP Kids program. Eligible participants were prospectively enrolled in the order in which they were referred, in an effort to create a sample largely reflective of those obese children who had not experienced success through initial office-based counseling alone. Upon referral, a pre-screening appointment was scheduled with the team social worker to assess eligibility, family needs, and commitment. Those aged 8–17 with primary obesity (BMI, calculated as weight in kilograms divided by height in meters squared, at or above the 95th percentile for age and sex, Centers for Disease Control and Prevention October 2000 growth charts), and who had at least one caregiver willing to participate in the program sessions were eligible for the study [13].

HIP Kids operated as an evening clinic to increase accessibility and provided individualized lifestyle education for participants and their families. The core multidisciplinary team consisted of a registered dietitian, exercise specialist, social worker, and pediatrician. The 1-year intervention consisted of an initial 3-month intensive phase with bi-weekly individual counseling sessions, followed by a 9-month maintenance phase with alternating monthly individual or group sessions. Each of the 15 sessions was 90 minutes in length and was divided into three 30-minute educational blocks focusing on nutrition, physical activity, and psychosocial and behavioral aspects. These educational blocks were led by a dietitian, exercise specialist, and social worker, respectively. A pediatrician performed the medical assessments and discussed the participant’s laboratory results at the initial and exit visits, and provided medical counseling when needed. Although it was only mandatory for the participant and one parent to attend, the participation of both parents, siblings, and other caregivers was continuously encouraged. Session attendance was recorded. Family involvement was central to the program design and included jointly developing strategies for attaining short and long-term goals, as well as the goals themselves. The strategies and skills required for a successful lifestyle modification which were discussed during sessions were based on social cognitive theory discussed by Bandura and on Epstein's model of family-based interventions, and included the following three key domains [14-16]: (i) nutrition (e.g., Canada’s Food Guide recommendations, food label reading, portion size, healthy recipes); (ii) physical activity (e.g., how to incorporate physical activity recommendations into daily life, make them fun, and involve the whole family, limiting screen time); and (iii) parenting/psychosocial factors (e.g., parenting styles, modeling and reinforcing desired behaviors, modifying the home environment). Discussion topics and strategies for overcoming barriers and for meeting previously set goals were all reassessed over the course of the intervention. Struggling families were continuously encouraged and received focused support directed at guiding them to overcome the challenges that had prevented them from achieving their goals. An intake visit was scheduled prior to the start of the program to review details of the program and to perform an initial study assessment (as described below) and a physical examination. Similarly, the exit visit at the end of the program incorporated final assessments and a physical exam, as well as program results, future goals, and participant feedback.

The study was approved by the University of Western Ontario Health Sciences Research Ethics Board and written informed consent was obtained from participating children and caregivers.

### Measures

All assessments were performed at the beginning and at the end of the program (baseline and 12 months). Additional anthropometric and body composition measures were obtained at 3 months, following the intensive phase of the program.

Height (to the nearest 0.1 cm) and weight (to the nearest 0.1 kg) were measured using standardized and calibrated equipment (stadiometer and scale, respectively), and were used to calculate BMI (BMI = weight/height^2^). BMI-Z was calculated using the U.S. Centers for Disease Control and Prevention reference data, which includes data for different ethnicities. Waist circumference (WC) was measured in the standing position midway between the iliac crest and the lowest rib to the nearest 0.1 cm using a constant tension tape. Body composition (percent body fat (BF%), fat-free mass (FFM)) was assessed through Bioelectrical Impedance Analysis (BIA) using a Tanita TBF-300 GS body composition analyzer (Tanita Corporation, Tokyo, Japan) [17]. The body composition of a subsample of patients was also measured through Dual energy X-ray absorptiometry (DXA) using a Hologic QDR 4500 (Hologic, Inc., Bedford, USA), to assess the correlation between BIA- and DXA-derived measurements [see Additional file 1].

Fasting glucose, insulin, LDL and HDL cholesterol, triglycerides, AST, and ALT were measured. A standard oral glucose tolerance test (oGTT) was used to assess glucose tolerance, with impaired glucose tolerance (IGT) defined as a 2-hour plasma glucose between 7.8 and 11.0 mmol/L and diabetes as a 2-hour plasma glucose ≥ 11.1 mmol/L. The Homeostasis Model Assessment (HOMA-IR = fasting plasma insulin (μU/mL) × fasting serum glucose (mmol/L)/22.5) was used to determine insulin resistance, defined as a HOMA ≥ 3.0 [18]. Hypertriglyceridemia was defined as triglycerides ≥ 1.50 mmol/L.

Three-day food records were used to assess dietary intake and eating patterns with “servings” defined according to Health Canada’s “Eating Well with Canada's Food Guide”. The Pediatric Quality of Life Inventory 4.0 (PedsQL) Child (age 8–12) and Teen (age 13–18) Report and Parent Reports were used to assess QoL and consisted of 23-item validated self-report questionnaires (maximum total score 100). Reported scores [SD] for Physical and Emotional Functioning in healthy children are 84.4 [17.3] and 80.9 [19.6] (Child Report), and 89.3 [16.4] and 82.6 [17.5] (Parent Report), respectively [19]. The Physical Activity Questionnaires for children and adolescents (PAQ-C, PAQ-A, ages 8–14 and >14, respectively) were used to assess physical activity behavior from the previous 7 days (minimum score 1, maximum score 5, indicating a low and high physical activity level) [20,21].

### Statistical analyses

Data were analyzed using IBM SPSS Statistics, version 20.0. Continuous variables were expressed as means and standard deviation (SD), and percentages were used to summarize categorical variables. Within-group differences were assessed using a paired sample *t*-test, and the 95% confidence interval was reported for the mean difference. Skewed continuous variables were reported as median and range, and the Wilcoxon signed-rank test was used to compare median differences between start and finish for skewed continuous variables. A P value < 0.05 was considered statistically significant. An a priori sample size calculation was not performed since this study aimed to pilot our intervention in a first sample of children to inform a subsequent larger trial.

## Results

**Figure 1:**
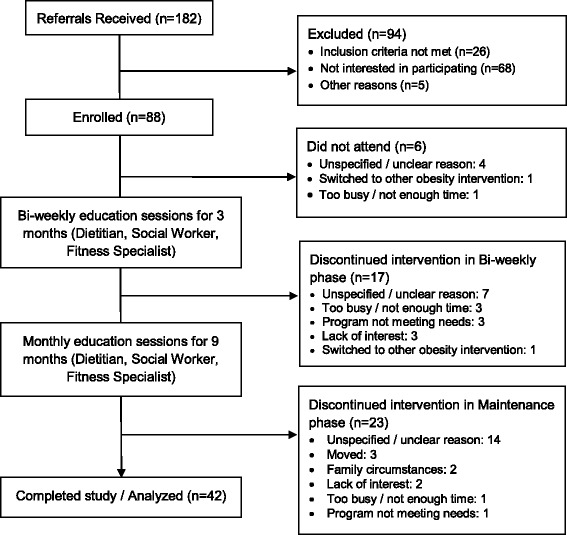
**Flow of subject progress from point of referral until the end of the 12-month intervention.**

Of the 88 childern who enrolled, 82 began the intervention. 17 left the study during the first phase of the program and 23 during the second phase (Figure 1). Data for the 42 children who completed the study (retention rate 47.7%) were included in the final analysis. Reasons given for leaving the intervention included: too busy (5), lack of interest (5), program not meeting needs (4), moving (3), while 25 families did not provide a reason for their discontinuation (Figure 1). Of those who withdrew, multiple participants verbally stated that they have found it challenging to build a trustful relationship with new members of the research team following changes in staff.

Of the 42 children (21 boys) who completed the study, mean age [SD] at baseline was 12.8 [3.14] years. 34 children (81%) were Caucasian, two (4.8%) were African American and Hispanic, respectively, one (2.4%) was of Aboriginal descent, and 3 (7.1%) were of Arabic descent. Mean Tanner Stage was 3, mean BMI [SD] was 31.96 [5.94] kg/m^2^, and mean BMI-Z was [SD] 2.19 [0.34]. QoL (specifically physical and emotional functioning) was impaired according to both the children’s and parental assessments, with mean baseline scores [SD] of 74.5 [16.5] and 63.7 [19.4] for physical functioning, and 69.0 [14.9] and 64.0 [18.3] for emotional functioning. 20 participants were insulin-resistant, 1 had IGT, 10 had acanthosis nigricans, and 11 had hypertriglyceridemia.

Mean baseline characteristics were similar between the study population and those who withdrew. A breakdown by gender, however, revealed that those who withdrew were more likely to be female and were, on average, 1.2 years older and more obese (higher BMI, % body fat, WC) than girls in the study group (Table 1).

**Table 1:** **Baseline characteristics of the total sample (Study population and Population that withdrew), separated by sex**

	**Study population (n = 42)**			**Population that withdrew (n = 46)**		
	**Total**	**Boys**	**Girls**	**Total**	**Boys**	**Girls**
n, No. (%)	42 (100)	21 (50.0)	21 (50.0)	46 (100)	16 (34.8)	30 (65.2)
Age, mean (SD), y	12.8 (3.14)	13.5 (2.59)	12.2 (3.49)	12.7 (2.95)	11.14 (2.11)	13.43 (3.02)
Body mass index, mean (SD), kg/m^2^	31.96 (5.94)	32.63 (5.97)	31.29 (5.67)	32.08 (6.49)	29.17 (6.88)	33.63 (5.69)
Body mass index z -score, mean (SD)	2.19 (0.34)	2.16 (0.34)	2.22 (0.34)	2.19 (0.32)	2.08 (0.35)	2.25 (0.29)
Waist circumference, mean (SD), cm	103.10 (14.66)	107.35 (15.47)	97.84 (11.60)	102.26 (16.32)	97.09 (16.78)	104.94 (15.41)
Body fat (BIA), mean (SD), %	40.95 (7.24)	39.96 (9.01)	41.95 (4.64)	41.89 (6.86)	37.26 (7.25)	44.87 (4.95)
Fat-free mass (BIA), mean (SD), kg	48.89 (15.00)	53.61 (16.89)	44.16 (10.94)	44.55 (10.67)	38.72 (7.70)	48.55 (7.45)
Tanner Stage, mean (SD)	3 (1.66)	3 (1.62)	3 (1.70)	3 (1.73)	2 (1.39)	4 (1.54)
QoL - Physical Functioning Score, Child report, mean (SD)	74.45 (16.47)	75.18 (13.19)	73.79 (18.93)	75.05 (15.89)	78.26 (10.84)	73.39 (17.73)
QoL - Physical Functioning Score, Parent report, mean (SD)	63.70 (19.43)	60.20 (20.90)	67.21 (17.13)	64.17 (17.86)	70.97 (13.23)	60.21 (18.97)
QoL - Emotional Functioning Score, Child report, mean (SD)	69.04 (14.86)	67.54 (15.00)	70.40 (14.59)	68.66 (17.16)	66.78 (16.28)	69.63 (17.52)
QoL - Emotional Functioning Score, Parent report, mean (SD)	64.02 (18.29)	68.48 (20.65)	70.34 (15.19)	63.19 (16.66)	65.70 (14.28)	61.73 (17.74)
Physical activity score, mean (SD)	2.26 (0.76)	2.40 (0.82)	2.12 (0.67)	2.47 (0.63)	2.69 (0.61)	2.37 (0.61)

Participants attended a mean [SD] of 75% [15.0] of all educational sessions. Following the first, more intensive program phase, BMI-Z slightly decreased (mean [SD] −0.04 [0.09]; 95% CI −0.07 to −0.02; P = .004), and BMI remained largely unchanged (mean [SD] -0.07 [1.01]; 95% CI −0.39 to 0.24; P = .66). Body composition did not change in a statistically significant way (Table 2). BMI-Z significantly decreased by the end of the 1-year program (mean [SD] -0.07 [0.11]; 95% CI −0.11 to −0.04; P = <.001). BMI increased (mean [SD] 0.80 [1.57]; 95% CI 0.31 to 1.29), but mean %BF and WC remained largely unchanged. FFM significantly increased (mean [SD] 4.02 [6.27] kg; 95% CI 1.90 to 6.14; P = <.001, Table 3).

**Table 2:** **Anthropometric and body composition changes from baseline to 3 months (end of the intensive program phase)**

	**Mean (SD) difference**	**95% CI**	***P*** **value**
	**(n = 41)**		
Height, cm	1.00 (1.07)	0.66 to 1.34	< 0.01
Body mass index, kg/m^2^	−0.07 (1.01)	−0.39 to 0.24	0.66
Body mass index z –score	−0.04 (0.09)	−0.07 to −0.02	< 0.01
Body fat (BIA), %	−1.39 (5.15)	−3.05 to 0.28	0.10
Fat-free mass (BIA), kg	0.99 (4.96)	−0.62 to 2.60	0.22

**Table 3:** **Changes in anthropometrics, body composition, quality of life, and physical activity from baseline to 12 months**

	**Mean (SD) difference**	**95% CI**	***P*** **value**
	**(n = 42)**		
Height, cm	4.25 (3.07)	3.29 to 5.20	< 0.01
Body mass index, kg/m^2^	0.80 (1.57)	0.31 to 1.29	< 0.01
Body mass index z-score	−0.07 (0.11)	−0.11 to −0.04	< 0.01
Waist circumference, cm	−0.31 (5.90)	−2.25 to 1.63	0.75
Body fat (BIA), %	−0.73 (5.18)	−2.48 to 1.03	0.41
Fat-free mass (BIA), kg	4.02 (6.27)	1.90 to 6.14	< 0.01
QoL - Physical Functioning Score, Child report	5.34 (17.12)	0.45 to 11.14	0.07
QoL - Physical Functioning Score, Parent report	11.34 (19.20)	4.74 to 17.94	< 0.01
QoL - Emotional Functioning Score, Child report	7.94 (14.32)	3.16 to 12.71	< 0.01
QoL - Emotional Functioning Score, Parent report	7.65 (15.69)	2.26 to 13.04	0.01
Physical Activity Score	0.22 (0.69)	−0.01 to 0.45	0.06

QoL parameters, expressed through the physical and emotional functioning scores, improved over the course of the study. Increases in both parent-reported scores (mean [SD] 11.3 [19.2]; 95% CI 4.7 to 17.9; P = .001, and mean [SD] 7.7 [15.7]; 95% CI 2.3 to 13.0; P = .007, respectively), and in the child-reported emotional functioning score (mean [SD] 7.9 [14.3]; 95% CI 3.2 to 12.7; P = .002) reached statistical significance (Table 3). The Physical Activity Score increased by 9.7%, reflecting a trend towards improvement (P = .06).

With regard to nutrition parameters, reported daily consumption of sugar-sweetened beverages decreased (P = .01), as did the intake of milk and alternative products (P = .05). None of the other nutrition or laboratory parameters assessed during the study showed any statistically significant improvements (Table 4).

**Table 4:** **Changes in laboratory and nutrition parameters from baseline to 12 months (end of program)**

**Laboratory values (n = 37)**	**Mean (SD) baseline**	**Mean (SD) 12 months**	**95% CI**	***P*** **value**
Insulin, pmol/L	110.90 (59.15)	135.51 (79.824)	−4.83 to 54.05	0.10
Glucose (fasting), mmol/L	4.91 (0.38)	5.04 (0.37)	0.01 to 0.24	0.04
HOMA-IR	3.50 (1.91)	4.31 (2.55)	−0.15 to 1.78	0.10
HDL-cholesterol, mmol/L	1.11 (0.26)	1.14 (0.30)	−0.03 to 0.08	0.36
LDL-cholesterol, mmol/L	2.33 (0.53)	2.41 (0.60)	−0.11 to 0.25	0.42
Triglycerides, mmol/L	1.26 (0.63)	1.28 (0.52)	−0.17 to 0.19	0.90
Aspartate aminotransferase, U/L	26.46 (7.15)	24.26 (10.65)	−5.39 to 0.99	0.17
Alanine aminotransferase, U/L	30.35 (16.00)	28.26 (21.82)	−9.06 to 4.88	0.55
Nutrition parameters (n = 30)				
Fruit & vegetables (servings/day)	4.45 (2.56)	5.22 (3.20)	−1.81 to 0.27	0.14
Grain products (servings/day)	6.36 (2.35)	5.75 (2.05)	−0.40 to 1.60	0.23
Milk & Alternatives (servings/day)	2.58 (1.22)	2.19 (1.16)	0.00 to 0.78	0.05
Meat & Alternatives (servings/day)	1.86 (0.86)	1.74 (1.01)	−0.27 to 0.50	0.55
Snack consumption (servings/day)	1.82 (0.77)	1.59 (0.95)	−0.14 to 0.60	0.17
Sugar-sweetened beverages (servings/day)^1^	0.33 (0.00 – 2.33)	0.00 (0.00 – 2.00)		0.01
Juice consumption (servings/day)^1^	0.33 (0.00 – 5.33)	0.33 (0.00 – 3.00)		0.77

^1^Results expressed as median (range) due to unequally distributed (skewed) values.

The total program delivery cost per participant was $3240 (CAD), based on material costs and 836 face-to-face and 418 indirect staff hours per year.

## Discussion

This study assessed whether the HIP Kids program prevented further increases in participants’ BMI and BMI-Z, and whether it improved QoL. During the initial phase of the intervention, the participants’ BMI-Z decreased and their BMI did not increase. During the second, less intensive 9-month maintenance phase, BMI-Z continued to decrease, but BMI increased. Participant growth may explain this discrepancy, where absolute BMI increased in these growing children due to an increase in absolute weight. Our findings that only FFM (but not % BF or WC) increased over the study period support this notion. Increases in muscle mass secondary to increased levels of physical activity may have also contributed to the observed increase in FFM and BMI. BMI-Z, a measure adjusted for height, age, and sex relative to a smoothed reference distribution, seems more fitting for taking such growth-related changes into account [22]. Additionally, although BMI and BMI-Z are known to correlate with adiposity and have been commonly used as primary outcome measures in lifestyle interventions, they have limitations. For example, neither parameter can differentiate between lean body mass and body fat. We included measures of body composition as secondary outcomes in our study to address this limitation. Furthermore, since the onset of puberty varies among children, BMI-Z may have decreased if the majority of study participants experienced an earlier growth spurt than their age-matched peers in the reference population. Data on growth spurt onset and peak growth velocity for the CDC reference population is not available to provide a comparison.

The BMI-Z of our study population decreased by 0.07 (mean; 95% CI −0.11 to −0.04). While this is a modest, albeit statistically significant decrease, it is in accordance with findings from recent meta-analyses of RCTs that showed that when pooled together, outpatient childhood obesity treatment programs produced a decrease in BMI-Z of 0.06 to 0.10 compared to control or minimal intervention groups [23,24].

Obese children and adolescents are highly at risk of developing significant impairments in QoL. Contributing factors include lower levels of self-esteem, self-confidence, and physical activity, bullying, and a higher risk of depression [6,7,25]. Longitudinal studies have also indicated that pediatric obesity both precedes and predicts the development of low self-esteem [7]. It is not surprising, then, that the baseline QoL scores of our study population indicated a lower QoL. This is consistent with cross-sectional results from Schwimmer et al., who used the same QoL assessment tool and found mean QoL scores between 60.9 and 71.0 in a cohort of more obese children, compared to scores between 80.9 and 92.5 in healthy children [26]. Our study demonstrated that the intervention led to a statistically significant improvement in QoL, with mean score increases ranging from 5.3 to 11.3. This adds to the limited data showing that multidisciplinary, family-centered, outpatient lifestyle interventions may improve QoL in obese children and adolescents [6,27,28].

With regard to effects on physical activity (PA), although the 9.7% increase in the PAQ score over the course of the program may be interpreted as a positive trend, it did not reach statistical significance. PAQ may be more sensitive at capturing moderate-to-vigorous PA and consequently less sensitive at capturing overall physical activity [21]. Measures based on self-reporting also have limitations such as potential recall bias, reporting inaccuracies based on other factors such as feeling ashamed for not reaching set goals or for poor adherence, or wanting to provide answers that are thought to be desired. The emotional state of the participant when filling in the questionnaire may also play a role. Therefore, the reported PA performed over the previous 7 days may not have been completely accurate. On the other hand, while counseling on how to achieve a more active lifestyle was a regular part of all educational sessions, actual moderate-to-vigorous intensity PA was only provided during group sessions. This may not have been enough to promote a significant day-to-day increase in higher-intensity PA in participants' daily routines.

With the exception of sugar-sweetened beverage (SSB) and milk and alternative product consumption, no statistically significant changes were seen in the laboratory and nutrition parameters assessed during the study. Improvements in insulin sensitivity through PA are well documented in the literature published on this topic. LeBlanc et al. also demonstrated a dose–response relationship between the amount of moderate-to-vigorous PA and improvements in lipid parameters [29]. It is therefore possible that a more pronounced increase in PA would have been necessary to produce a positive effect on the laboratory-based outcomes. A greater reduction in BF may have also contributed to an improved insulin sensitivity and lipid profile.

The observed decrease in SSB consumption must be interpreted with caution. Not only were the values skewed, but the reported intake both at baseline and especially at 12 months was much lower than values seen in the literature for this population, which may indicate underreporting [30].

Our study possesses several strengths, including a multidisciplinary, family-centered design according to evidence-based recommendations [12,31]. Program session frequency (bi-weekly to monthly) was also consistent with findings from Hampl et al., whose study showed that 74% of the 24 U.S. pediatric obesity clinics and 78% of the 24 U.S. pediatric obesity programs included in their analysis offered these session intervals [32]. Conducting the majority of program sessions as individual sessions also allowed us to tailor counseling and goal setting according to each family’s needs and their specific obesity-related triggers.

There is still little data on the impact of obesity interventions on QoL in children and adolescents and a need for more research in this area has been identified [6]. Our results add to the limited data that indicate that multidisciplinary obesity interventions can have a positive impact on QoL and provide more detailed insight into specific QoL domains using a validated and recognized tool.

Furthermore, given the current prevalence of childhood obesity, generating information on related health care costs is becoming increasingly important. Therefore, our data on program delivery costs may assist others in determining program budgets and in calculating health care costs for this type of study population.

This study also has several limitations. As a pilot study designed to meet the clinical needs of our local population, this phase of HIP Kids was not designed as a controlled trial. Since an a priori sample size calculation was not performed, non-significant findings may have been the result of inadequate statistical power. The attrition rate of 52% was also somewhat higher than the up to 42% reported in recent meta-analyses for RCTs examining pediatric obesity interventions and reporting dropout rates [10]. Of note, most of the analyzed studies did not adequately report attrition. Given that two-thirds of those who withdrew from our study were adolescent girls, HIP Kids may not have properly met the needs of female participants. Tailoring the program more specifically to the different needs and expectations of adolescent boys and girls may have reduced attrition. This would be in keeping with data by Knöpfli et al., who demonstrated significant gender-related differences in intervention outcomes favoring boys in a larger inpatient cohort [33]. Other potential contributing factors may have included a lack of standardized motivational pre-screening, accepting participants living up to 1.5 hours driving distance away, a hospital- rather than a community-based program location, and the decreased frequency of sessions during the 9-month maintenance phase of the program. The latter may not have been sufficient to keep participants motivated and committed. Also, staff changes during the course of the program may have affected the ability of some participants to maintain a trustful relationship with the team and may have contributed to the observed attrition. Still, a thorough interview process and mandatory standardized training of all newly hired personnel had been in place to minimize the impact of staff changes on attrition.

## Conclusion

In an era when obesity has become one of the most prevalent health concerns for children and adolescents, there is still a need for more data to determine the ideal design and the effectiveness of treatment interventions for this condition. This pilot study served to create and assess a program based on the 2008 PCCH recommendations in southwestern Ontario, Canada, where none existed before, and to estimate the costs associated with providing such an intervention.

The results of our study indicate that although the HIP Kids multidisciplinary lifestyle intervention program did not prevent the participants’ raw BMI score from increasing over the course of the intervention, it reduced participants’ BMI-Z scores, improved health-related QoL outcomes and prevented further increases in other measures of adiposity (% BF, WC). Our results also suggest that raw BMI may not correlate strongly enough with changes in adiposity to be a useful primary outcome parameter for determining the effectiveness of pediatric obesity intervention studies. Future research into the development of effective intervention strategies in the studied population should focus on the following: (i) generating more data on health-related QoL outcomes; (ii) examining whether a community setting is more effective than a hospital setting and if this leads to a higher retention rate; and (iii) tailoring interventions to the gender-specific needs of obese adolescents. Lastly, to further improve retention, interventions should include a standardized, motivational pre-screening tool (a “Readiness to Change” assessment), and researchers may consider defining a maximal residential radius from the program site in their inclusion criteria.

## Additional file

Additional file 1: Table S1. Correlation between BIA- and DXA-derived body composition measures at baseline and 12 months in a subsample of participants. This data shows our correlation analysis of body composition measures derived by BIA and DXA in a subsample of participants and demonstrates that the two compared methods very highly correlate for measuring fat-free mass and highly correlate for measuring body fat.

## Abbreviations

BF%: Percent body fat
BMI: Body mass index
BMI-Z: BMI z-score
FFM: Fat-free mass
IGT: Impaired glucose tolerance
oGTT: Oral glucose tolerance test
PAQ-C/PAQ-A: Physical Activity Questionnaire for children/for adolescents
PCCH: Provincial Council for Children’s Health
PedsQL: Pediatric Quality of Life Inventory 4.0
QoL: Quality of Life
WC: Waist circumference
