# Supplementary material for: The Health Initiative Program for Kids (HIP Kids): effects of a 1-year multidisciplinary lifestyle intervention on adiposity and quality of life in obese children and adolescents - a longitudinal pilot intervention study
Source: BMC Pediatr. 2014 Dec 5;14:296. doi: 10.1186/s12887-014-0296-1 (PMC4263062; doi:10.1186/s12887-014-0296-1)
Supplement: Additional file 1: Table S1. — Correlation between BIA- and DXA-derived body composition measures at baseline and 12 months in a subsample of participants. This data shows our correlation analysis of body composition measures derived by BIA and DXA in a subsample of participants and demonstrates that the two compared methods very highly correlate for measuring fat-free mass and highly correlate for measuring body fat. [file 12887_2014_296_MOESM1_ESM.docx]

**Additional file 1: Table S1: Correlation between BIA- and DXA-derived body composition measures at baseline and 12 months in a subsample of participants.**

| Measures | Correlation coefficient | *P* Value |
| --- | --- | --- |
|  | n = 24 (baseline), n = 15 (12 months) | |
| Body fat (baseline), %: BIA versus DXA | 0.76 | < 0.01**^*^** |
| Fat-free mass (baseline), kg: BIA versus DXA | 0.95 | < 0.01**^*^** |
| Body fat (12 month), %: BIA versus DXA | 0.88 | < 0.01**^*^** |
| Fat-free mass (12 month), kg: BIA versus DXA | 0.96 | < 0.01**^*^** |

**^*^**Correlation is significant at the 0.01 level (2-tailed).
